# Primary gastric SMARCA4-deficient carcinosarcoma and sarcomatoid carcinoma: Two case reports

**DOI:** 10.3389/fonc.2025.1663811

**Published:** 2025-10-15

**Authors:** Ran Zhao, Jun jun Zhang, Xiao Zhi, Ren ya Zhang, Lei Li

**Affiliations:** Department of Pathology, Affiliated Hospital of Jining Medical University, Jining, Shandong, China

**Keywords:** gastric tumor, SMARCA4-deficient, carcinosarcoma, sarcomatoid carcinoma, case report

## Abstract

Gastric SMARCA4-deficient carcinosarcoma and sarcomatoid carcinoma are rare with poor prognosis. In the present study, two male patients were hospitalized due to abdominal manifestations, specifically abdominal pain and a sense of fullness and discomfort. Gastroscopic examination revealed huge raised lesions in the stomach of both individuals, subsequently leading to laparoscopic radical total gastrectomy. Histopathological analysis of surgical specimens obtained from both patients demonstrated the presence of carcinomatous and sarcomatous components. In Case 1, the sarcoma region was negative for the SMARCA4, epithelial markers cytokeratin (CK) and epithelial membrane antigen (EMA), CK, EMA, and SMARCA4 were positively expressed in the adenocarcinoma area, thereby warranting carcinosarcoma diagnosis. The sarcomatous component was identified as a tumor characterized by SMARCA4 deficiency. In Case 2, the tumor cells exhibited positive staining for CK and Vimentin, negative staining for SMARCA4, and the patient was positive for SMARCA4-deficient sarcomatoid carcinoma.

## Introduction

SMARCA4 mutations occur in 7% of human tumors (1) and affect multiple organ systems. The mutation frequency of SMARCA4 varies significantly across different tumor types. It has been reported to be mutated in 15% of Burkitt’s lymphoma (2), 10-35% of non-small-cell lung carcinoma (3, 4), 5-10% of medulloblastoma and melanoma (5–7). The mutation rate of SMARCA4 in gastric cancer is relatively low, approximately 2% (8).SMARCA4-deficient tumors are typically poorly differentiated or undifferentiated, exhibit highly malignant and invasive characteristics, and show resistance to chemotherapy (4). Histologically, adenocarcinoma is the most frequently observed cancer (8), while sarcomatoid and carcinosarcomas variants are uncommon.

This study presents two cases of SMARCA4-deficient gastric tumors: one case of carcinosarcoma and one of sarcomatoid carcinoma. It elaborates on their histological similarities and differences and further explores potential therapeutic approaches for SMARCA4-deficient gastric tumors. The main clinicopathologic characteristics of these two patients are summarized in **Table 1**.

**Table 1:** The clinicopathologic features of gastric SMARCA4-deficient carcinosarcoma and sarcomatoid carcinoma.

Clinical date	Case1	Case2
Age (years)/sex	50/male	68/male
Symptoms	Upper abdominal fullness and discomfort	Abdominal pain and belching
Location	Gastric fundus	Gastric fundus on the lesser curvature
Size	6 × 7 cm	10 x 8 cm
CT	Thickening of the gastric wall; mild enhancement	Thickening of the gastric wall; mild-to-moderate heterogeneous enhancement
Endoscopic finding	Protruding lesion; ulcerative	Protruding lesion; ulcerative
Borrmann	Type I	Type I
Pathologic diagnosis	SMARCA4-deficient Carcinosarcoma	SMARCA4-deficient sarcomatoid carcinoma
Histologic features	Moderately differentiated; glandular; rhabdoid	undifferentiated; rhabdoid
SMARCA4 expression	Positive in the adenocarcinoma, total loss in the sarcoma	total loss
TNM stage	pT4N2M0	pT4N1M0
Therapeutic approach	Resection	Resection; traditional Chinese medicine
Recurrence	liver	no
Duration after surgery	24M	25M
outcome	dead	alive

## Case presentation

### Case1

**Figure 1:**
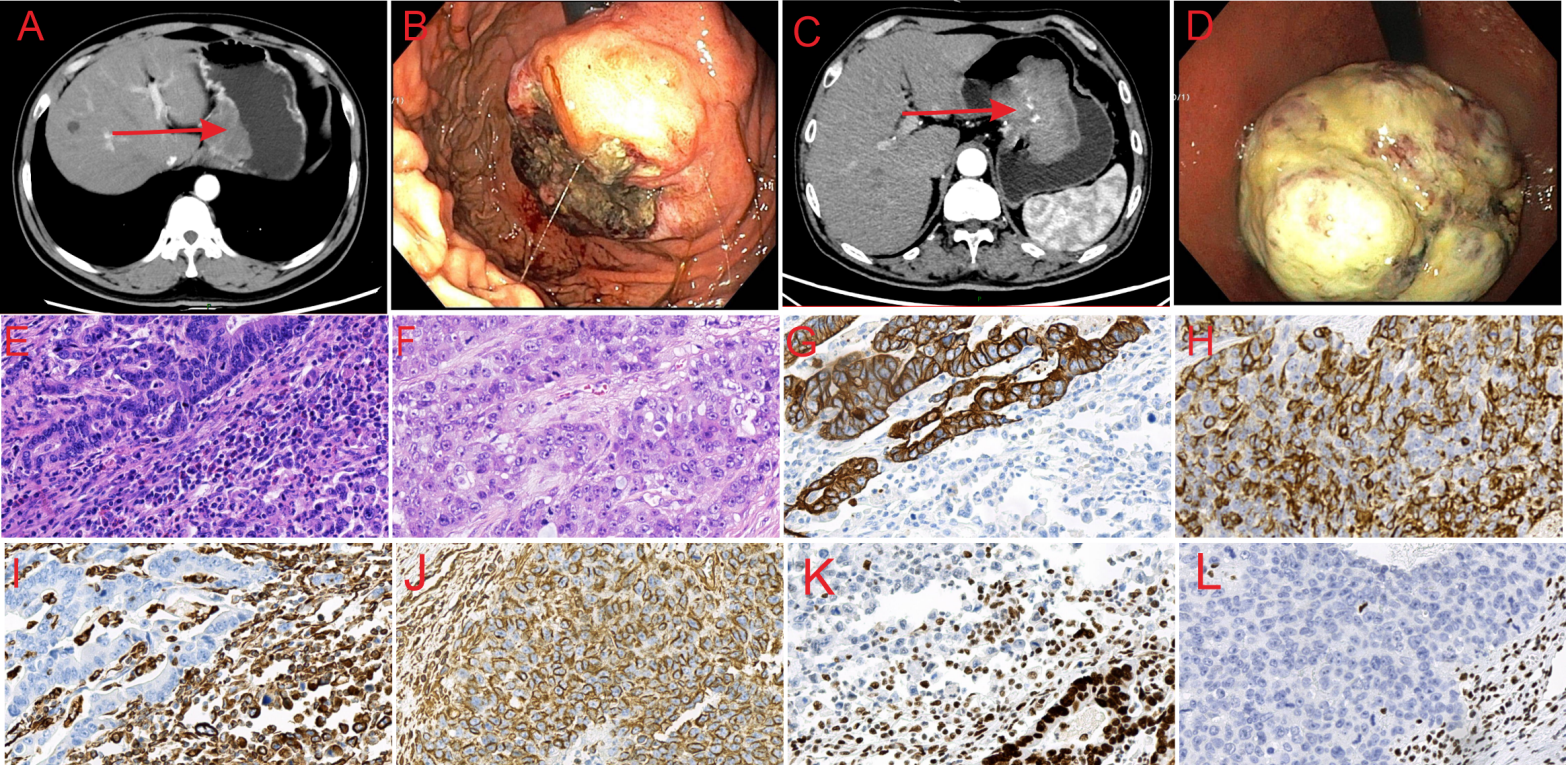
Abdominal CT, endoscopic and histological features of the lesions. **(A)** CT image of SMARCA4-deficient carcinosarcoma showing thickening of the lower esophageal segment and gastric cardia wall. **(B)** CT image of SMARCA4-deficient sarcomatoid carcinoma showing thickening of the gastric lesser curvature and side-cardia wall, with an irregular mass shadow. **(C)** Endoscopic image of SMARCA4-deficient carcinosarcoma showing a large, protruding lesion on the gastric fundus mucosa, with a ruptured surface. **(D)** Endoscopic image of SMARCA4-deficient sarcomatoid carcinoma showing a large, protruding lesion near the gastric fundus on the lesser curvature. **(E)** Microscopic image of SMARCA4-deficient carcinosarcoma (HE, ×40). **(F)** Microscopic image of SMARCA4-deficient sarcomatoid carcinoma (HE, ×40). **(G)** CK staining shows positive expression in the adenocarcinoma area and negative expression in the sarcoma area of SMARCA4-deficient carcinosarcoma (×40). **(H)** CK staining showing positive expression in SMARCA4-deficient sarcomatoid carcinoma (×40). **(I)** Vimentin staining showed negative expression in the adenocarcinoma area and positive expression in the sarcoma area of SMARCA4-deficient carcinosarcoma (×40). **(J)** Vimentin staining showing positive expression in SMARCA4-deficient sarcomatoid carcinoma (×40). **(K)** SMARCA4 staining showing positive expression in the adenocarcinoma area and negative expression in the sarcoma area of SMARCA4-deficient carcinosarcoma (×40). **(L)** SMARCA4 staining showing negative expression in SMARCA4-deficient sarcomatoid carcinoma (×40).

A 50-year-old male patient was admitted to the hospital for upper abdominal fullness and discomfort lasting 6 months and difficulty in swallowing for more than 20 days. The patient had no specific medical or family history. Physical examination revealed deep tenderness in the upper abdomen. The serum tumor-related antigen was 99.47 U/mL (normal, 0–95 U/mL); all other laboratory results were normal. Enhanced computed tomography (CT) showed thickening of the gastric cardia wall in the lower esophageal segment with mild enhancement (**Figure 1A**); multiple hepatic cysts. Gastroscopy revealed a large protruding lesion on the gastric fundus mucosa with a ruptured surface, Borrmann type I (**Figure 1C**). Four biopsy specimens were taken from the edge of the ulceration. The biopsy pathology was diagnosed as tumor with SMARCA4 deficiency. Immunohistochemistry result: HMB-45(-), S-100(-), EMA(-), CAM5.2(-), ERG(-), INI-1(+), CK局灶(+), CK7(-), CK5/6(-), p40(-), Vimentin(+), SMARCA4(-), Ki-67(+,70-80%).

The patient underwent radical total gastrectomy with esophagojejunostomy, but declined postoperative adjuvant treatment. Lymph nodes no. 1, 2, 3, 5, 6, 7, 8, 9 and 11 were removed during the operation. The tumor measured approximately 7 cm × 6 cm. Histopathological analysis revealed a mixed tumor composed of moderately differentiated adenocarcinoma and sarcoma. In the adenocarcinoma region, cancer cells formed glandular tubular structures with fibrinoid necrosis. The boundary between the adenocarcinoma and sarcomatous areas was unclear. Sarcomatous cells were loosely arranged, spindle-shaped, uniform in size, and displayed bizarre nuclei, numerous mitotic figures, rich cytoplasm, eccentric nuclei, large nuclei, obvious nucleoli, and many inflammatory cell infiltrations (**Figure 1E**). The tumor invaded the entire thickness of the gastric wall and the nerve. Cancer emboli were observed in the vessels. There were three lymph node metastases: one was an adenocarcinoma, and two were sarcoma components. Immunohistochemistry revealed that CK (**Figure 1G**), EMA, and SMARCA4 (**Figure 1K**) were positively expressed in the adenocarcinoma area, whereas Vimentin (**Figure 1I**) was negative; CK (**Figure 1G**), EMA, SMARCA4 (**Figure 1K**), ERG, Desmin, CD34, HMB-45, S100, CK7, CK5/6, and p40 were negatively expressed in the sarcoma area, while Ki-67(+50–60%), Vimentin (**Figure 1I**), and INI-1 were positively expressed. The final diagnosis was carcinosarcoma with a SMARCA4 deficiency in the sarcoma area; pT4N2M0.

**Figure 2:**
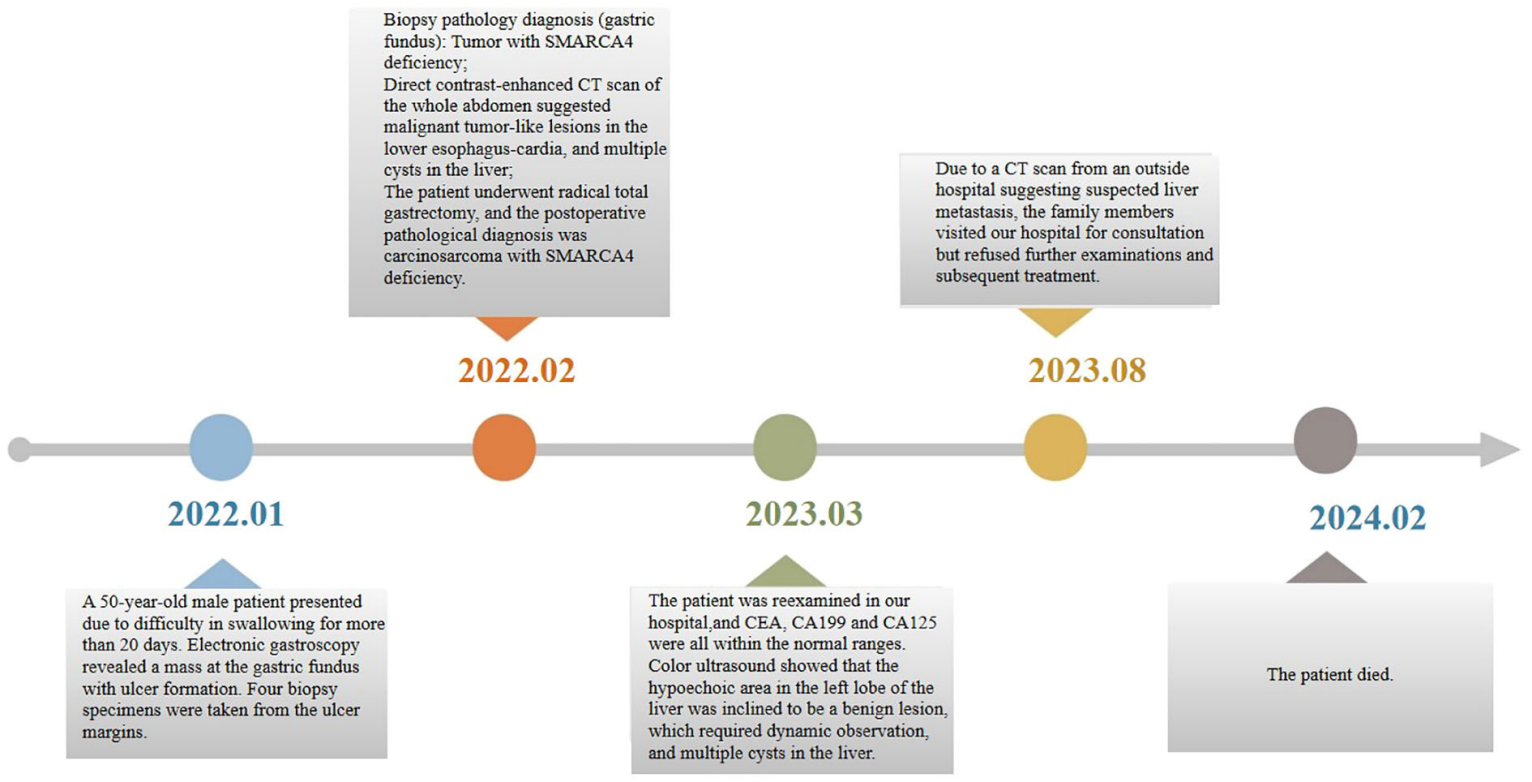
The consultation timeline of patients with SMARCA4-deficient carcinosarcoma.

The patient underwent a re-examination 12 months after surgery. CEA, CA199, and CA125 were all within the normal ranges. Color ultrasound showed that the hypoechoic area in the left lobe of the liver was inclined to be a benign lesion, which required dynamic observation, and multiple cysts in the liver. Eighteen months after surgery, the patient was readmitted to hospital for liver metastasis detected by a CT scan at an out-of-town hospital, but refused further examinations and treatment. The patient ultimately survived for 24 months. The patient’s timeline of consultations is shown in **Figure 2**.

### Case2

A 68-year-old male patient was admitted to the hospital for abdominal pain and belching lasting over 1 month. The patient had no specific medical or family history. Laboratory tests showed normal levels of CA19-9, carcinoembryonic antigen, and alpha-fetoprotein. Enhanced CT revealed thickening of the gastric lesser curvature at the cardia wall, with an irregular mass, and mild-to-moderate heterogeneous enhancement (**Figure 1B**). Gastroscopy showed a large protruding lesion near the gastric fundus on the lesser curvature, with ulceration on the oral side, Borrmann type I (**Figure 1D**). Six biopsy specimens were taken from the oral side. The biopsy pathology was diagnosed as adenocarcinoma of the gastric body with necrosis. The patient underwent radical total gastrectomy with esophagojejunostomy. Perigastric lymph nodes were removed during the operation.

The tumor measured approximately 10 cm × 8 cm. Microscopically, the tumor cells were arranged disorderly, without the structural hierarchy of normal tissues. They contained cancerous and sarcomatoid components. The cancerous component contained moderately differentiated epithelial-like nest cells, while the sarcomatoid component featured diffuse spindle growth cells invading the entire thickness of the gastric wall (**Figure 1F**). Cancer emboli were detected in the vessels. CK (**Figure 1H**), Vimentin (**Figure 1J**) and Ki-67 (>75%) were positive in the tumor cells, whereas SMARCA4 (**Figure 1L**), CD20, LCA, CD3, HMB-45, Melan-A, Epstein-Barr virus (EBV) were negative. Mismatch repair proteins were intact. The final diagnosis was SMARCA4-deficient sarcomatoid carcinoma; pT4N1M0.

**Figure 3:**
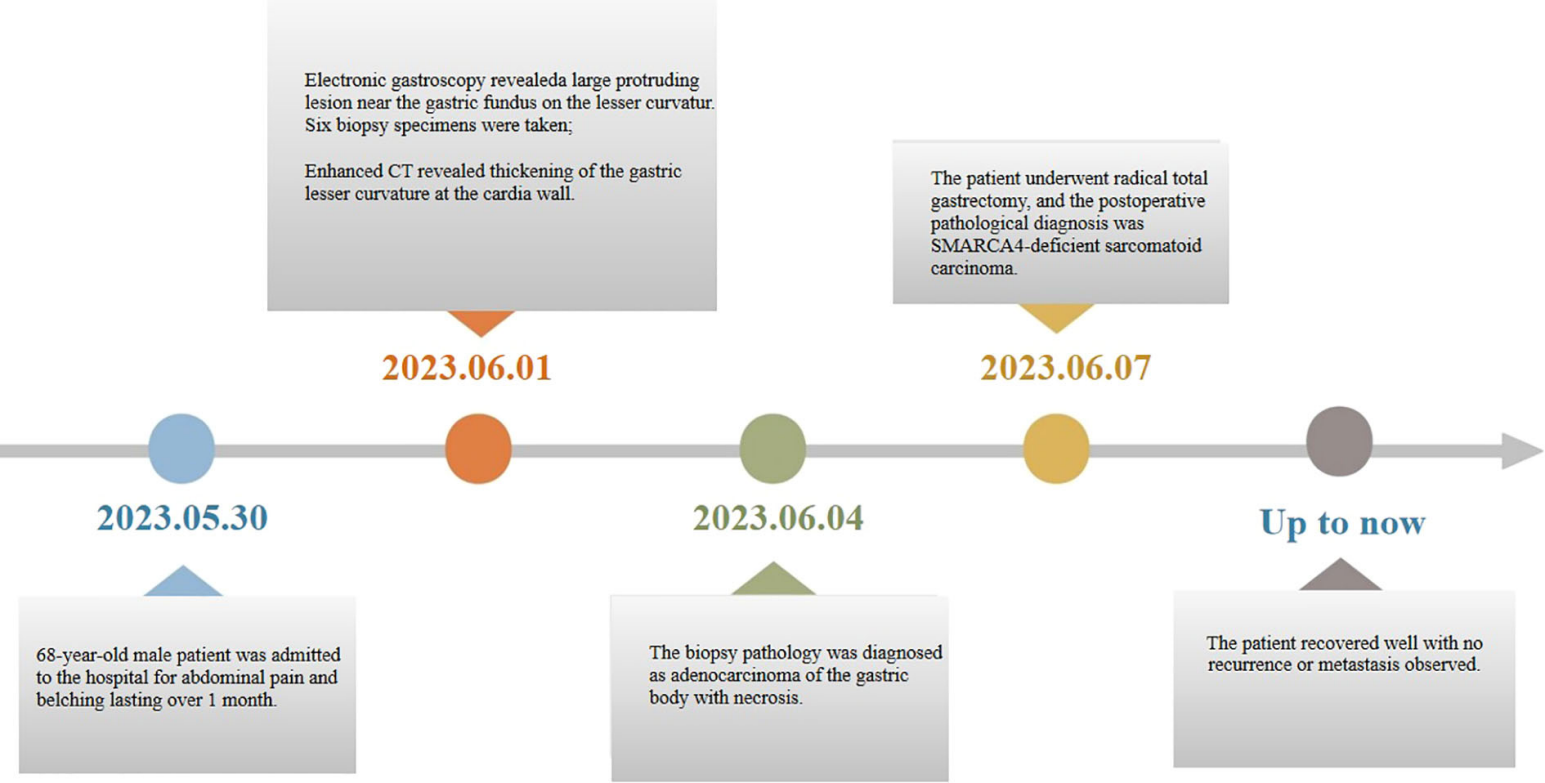
The consultation timeline of patients with SMARCA4-deficient sarcomatoid carcinoma.

After surgery, the patient refused chemotherapy and received traditional Chinese medicine (TCM) treatment at another hospital, with no recurrence or metastasis, during the 25-month telephone follow-up. The patient’s timeline of consultations is shown in **Figure 3**.

## Discussion

The SWitch/sucrose non-fermentable (SWI/SNF) chromatin remodeling complex is a multi-protein complex consisting of 10–15 subunits, regulates gene activity through chromatin remodeling processes, maintains genomic stability, and controls cell growth, differentiation, and other biological behaviors. SMARCA4 is the primary SWI/SNF complex subunit that generates energy through the catalysis of ATP hydrolysis, which is essential for ensuring the normal function of the complex, thereby maintaining the normal cell physiological function and gene expression patterns (9).

SMARCA4 mutations occur in almost all small cell carcinoma of the ovary hypercalcemic type (10) and 8% of non-small cell lung cancer cases (3). Gastric SMARCA4-deficient carcinosarcoma and sarcomatoid carcinoma are particularly rare. In the literature review, we identified only 4 previously reported cases of SMARCA4-deficient gastrointestinal tumors with sarcomatoid components that had a definitive pathological diagnosis. These cases were designated as carcinosarcomas or sarcomatoid carcinomas (11–13). A summary of these cases is provided in **Table 2**. The patients ranged in age from 46 to 73 years (mean, 66.3 years) with a male-to-female ratio of 3:1. The tumors were located at the esophagogastric junction (1 case), rectum (1 case), and colon (2 cases), with sizes ranging from 3.5 to 17 cm. Including the 2 cases reported in the present study, 5 out of 6 patients underwent surgical resection, among whom 2 received other postoperative adjuvant therapies and achieved favorable outcomes. Detailed information is presented in **Table 2**. SMARCA4-deficient tumors with sarcomatoid components in the chest and rectum (12, 14) are often associated with claudin-4 and SMARCA2 deficiencies; they had larger primary tumor volumes and shorter survival times than other types of SMARCA4-deficient tumors.

**Table 2:** Reported cases: clinicopathologic features of SMARCA4-deficient gastrointestinal tumors with sarcomatoid components.

NO.	References	Age (years) sex	Symptoms	Location	Size	CT	Endoscopic finding	Pathologic diagnosis	SMARCA4 expression	TNM stage	Therapeutic approach	Outcome (month)
1	(11)	73/male	dysphagia, odynophagi	gastro-esophageal junction and gastric fundus	17cm	abnormal wall thickening in the proximal stomach	a gastric mass	SMARCA4-deficient sarcomatoid tumor	total loss	T4aN2M0	Preoperative chemotherapy; resection	NA
2	(12)	46/male	a prolapsing anal mass	lower rectum	3.5x3cm	localized wall thickness	an irregular nodular elevated lesion	SMARCA4−deficient rectal carcinoma with a sarcomatoid component	total loss	T3N2bM0	Resection; chemotherapy; radiation therapy	12/alive
3	(13)	79/female	NA	Transverse	NA	NA	NA	SMARCA4-deficient sarcomatoid carcinoma	total loss	NA	NA	0/alive with disease in hospice
4		67/male	NA	Sigmoid	4.5cm	NA	NA	SMARCA4-deficient sarcomatoid carcinoma	SMARCA4-deficient sarcomatoid carcinoma	T4N2aM1b	Resection	1/dead

Carcinosarcomas and sarcomatoid carcinomas share some morphological overlap; however, they are distinct tumor entities (15). The SMARCA4-deficient carcinosarcoma reported in this study is an unclassified pleomorphic sarcoma, which is immunoreactive to the stromal marker Vimentin but non-immunoreactive to the epithelial markers CK, EMA. The cancerous component is a moderately differentiated adenocarcinoma. There is no transitional zone between the two components. The SMARCA4-deficient sarcomatoid carcinoma is essentially a special type of epithelial-derived carcinoma, in which tumor cells undergo mesenchymal-like morphological transformation. Tumor cells express CK and Vimentin. The cancerous component manifests as nests of epithelial-like cells, and there is a distinct transitional zone between the two components.

The cases described in this study require differentiation from malignant rhabdoid tumors, lymphomas, epithelioid gastrointestinal stromal tumors, melanomas, undifferentiated gastric carcinomas, NUT midline carcinomas, and other neoplastic entities. Most of these differential diagnoses can be accomplished using specific immunohistochemical markers or molecular detection approaches. For example, malignant rhabdoid tumors predominantly affect infants and young children, primarily arising in the central nervous system or soft tissues, with occasional involvement of the gastrointestinal tract. Immunohistochemically, the tumor cells exhibit expression of Vimentin, CKpan, and EMA. Lymphoma cells characteristically express lymphoid markers such as LCA. Epithelioid gastrointestinal stromal tumor cells are arranged in a nest-like or sheet-like pattern with epithelioid morphology, and their identification can be confirmed by positive immunohistochemical staining for CD117 and DOG1. Melanomas show positivity for immunohistochemical markers including HMB45, MelanA, and S100. As an epithelial-derived neoplasm, undifferentiated gastric carcinoma typically expresses epithelial markers and generally lacks or rarely exhibits expression of mesenchymal markers (e.g., Vimentin). In NUT midline carcinoma, abrupt foci of squamous differentiation are frequently observed, and the tumor cells harbor detectable BRD-NUT fusion and positive NUT expression.

Surgical resection remains the main treatment for SMARCA4-deficient tumors. However, it is still necessary to combine other adjuvant therapies to delay the progression of the disease. SMARCA4/2 deficiency reportedly restricts the flow of calcium ions mediated by IP3R3 from cancer cells to the mitochondria, leading to resistance to chemotherapeutic drugs (16). So most patients show poor responsiveness to chemotherapy (17). For example, a case report of a 73-year-old male with gastric SMARCA4-deficient high-grade sarcomatoid carcinoma showed that the patient was unresponsive to chemotherapy (11); however, a case report of a 59-year-old male with gastric SMARCA4-deficient sarcoma demonstrated that the patient had a good response to combined chemotherapy with doxorubicin and ifosfamide after surgery and remained alive during 49 months of follow-up (18). Both patients in this article refused postoperative chemotherapy, making it impossible to evaluate the efficacy of chemotherapy.

As a homologous subunit of SMARCA4 in the SWI/SNF complex, SMARCA2 has emerged as an attractive therapeutic target for SMARCA4-deficient tumors through a synthetic lethal effect (19). However, SMARCA2 is also mutated or inactivated in a variety of solid tumors. Previous studies have documented SMARCA2 mutations or deletion in 5-10% of non-small-cell lung carcinoma (20, 21), 10% of esophageal adenocarcinoma (22), 5-9% of gastric cancer (23, 24), 1.3% of colorectal carcinoma (25), 3% of clear cell renal cell carcinoma (26), and 5% adenoid cystic carcinomas (27). Notably, the loss of SMARCA2 has been observed in some tumors with SMARCA4 deficiency, such as 90-100% of small cell carcinoma of the ovary (hypercalcemic type) (28, 29), 67-80% of thoracic SMARCA4-deficient undifferentiated tumors (14, 30), 15-30% of SMARCA4-deficient non-small cell lung cancer (20, 21), 11% of SMARCA4-deficient gastric adenocarcinoma (23), 15% of SMARCA4-deficient colorectal cancer (25), 70% of undifferentiated endometrial carcinoma (31). This phenomenon limits the application of SMARCA2-targeted synthetic lethal therapies. SMARCA2-induced synthetic lethality also requires the complete loss of SMARCA4 function (1) and is ineffective against tumors with reduced or heterogeneous SMARCA4 expression. Second-generation histone deacetylase inhibitors (HDACis) can reactivate the expression of SMARCA2-dependent IP3R3, reduce the limitation of IP3R3-mediated calcium ion flow to mitochondria caused by SMARCA4/2 deficiency, enhancing their response to chemotherapy (16). However, they are unsuitable for SMARCA4-deficient tumors without SMARCA2 deletion. SMARCA4-deficient lung and ovarian cancer cells show increased sensitivity to the KDM6 inhibitor GSK-J4, which can be an effective treatment option after cisplatin resistance (32). However, its effect on gastric cancer cells is unknown. Programmed death-ligand *1* (PDL*1*) immunotherapy remains the most effective therapeutic option and is not restricted by PDL*1* expression levels (33). In our study, TCM treatment after surgery has led to no recurrence or metastasis during the 25-month follow-up period. It indicates that TCM treatment can become another widely applicable postoperative adjuvant treatment method.

This study provides a comprehensive description of two rare cases of primary gastric SMARCA4-deficient carcinosarcoma and sarcomatoid carcinoma. Histologically, the difference was the distinct responses of the sarcomatous components to epithelial markers. TCM treatment may serve as a post-surgical treatment option. However, due to the small sample size in this study, further studies are needed to better characterize these two tumor subtypes and their response to chemotherapy.

## Data Availability

The datasets presented in this study can be found in online repositories. The names of the repository/repositories and accession number(s) can be found in the article/supplementary material.
